# Transcutaneous auricular vagus nerve stimulation may benefit from the addition of N-acetylcysteine to facilitate motor learning in infants of diabetic mothers failing oral feeds

**DOI:** 10.3389/fnhum.2024.1373543

**Published:** 2024-05-22

**Authors:** Dorothea D. Jenkins, Sandra S. Garner, Alyssa Brennan, Jessica Morris, Kate Bonham, Lauren Adams, Sally Hunt, Hunter Moss, Bashar W. Badran, Mark S. George, Donald B. Wiest

**Affiliations:** ^1^Department of Pediatrics, Medical University of South Carolina, Charleston, SC, United States; ^2^Department of Clinical Pharmacy and Outcomes Sciences, College of Pharmacy, Medical University of South Carolina, Charleston, SC, United States; ^3^Department of Obstetrics and Gynecology, Medical University of South Carolina, Charleston, SC, United States; ^4^Department of Pediatrics, University of North Carolina, Chapel Hill, NC, United States; ^5^Department of Neuroscience and the Center for Biomedical Imaging, Medical University of South Carolina, Charleston, SC, United States; ^6^Neuro-X Lab, Department of Psychiatry, Medical University of South Carolina, Charleston, SC, United States; ^7^Brain Stimulation Division, Department of Psychiatry, Medical University of South Carolina, Charleston, SC, United States; ^8^Ralph H. Johnson VA Medical Center, Charleston, SC, United States

**Keywords:** taVNS, nasogastric N-acetylcysteine, glutathione, oxidative stress, poor feeding, infants, pharmacokinetics

## Abstract

**Objective:**

This study aims to determine if pretreating with enteral N-acetylcysteine (NAC) improves CNS oxidative stress and facilitates improvement in oromotor skills during transcutaneous auricular nerve stimulation (taVNS) paired with oral feedings in infants of diabetic mothers (IDMs) who are failing oral feeds.

**Methods:**

We treated 10 IDMs who were gastrostomy tube candidates in an open-label trial of NAC and taVNS paired with oral feeding. NAC (75 or 100 mg/kg/dose) was given by nasogastric (NG) administration every 6 h for 4 days, then combined with taVNS paired with 2 daily feeds for another 14 days. NAC pharmacokinetic (PK) parameters were determined from plasma concentrations at baseline and at steady state on day 4 of treatment in conjunction with magnetic resonance spectroscopic (MRS) quantification of CNS glutathione (GSH) as a marker of oxidative stress. We compared increases in oral feeding volumes before and during taVNS treatment and with a prior cohort of 12 IDMs who largely failed to achieve full oral feeds with taVNS alone.

**Results:**

NAC 100 mg/kg/dose every 6 h NG resulted in plasma [NAC] that increased [GSH] in the basal ganglia with a mean of 0.13 ± 0.08 mM (*p* = 0.01, compared to baseline). Mean daily feeding volumes increased over 14 days of NAC + taVNS compared to the 14 days before treatment and compared to the prior cohort of 12 IDMs treated with taVNS alone. Seven IDMs reached full oral feeds sufficient for discharge, while three continued to have inadequate intake.

**Conclusion:**

In IDM failing oral feeds, NAC 100 mg/kg/dose every 6 h NG for 4 days before and during taVNS paired with oral feeding increased CNS GSH, potentially mitigating oxidative stress, and was associated with improving functional feeding outcomes compared to taVNS alone in a prior cohort. This represents a novel approach to neuromodulation and supports the concept that mitigation of ongoing oxidative stress may increase response to taVNS paired with a motor task.

## 1 Introduction

Neonates and infants with brain injury of prematurity or hypoxic–ischemic encephalopathy (HIE) commonly have problems learning the oromotor skills of sucking, swallowing, and breathing and may require a gastrostomy tube (G-tube) for feeding. taVNS may have similar effects as implanted vagus nerve stimulation when paired with an activity, modulating neurotransmitter activity and inducing cortical and white matter neuroplasticity, boosting long-term potentiation and motor learning (Khodaparast et al., 2013; Capone et al., 2017; Dawson et al., 2021). In our first-in-infants trial, we enrolled 35 infants with feeding failure whose parents were in discussions about G-tube placement and paired transcutaneous auricular vagus nerve stimulation (taVNS) with the motor activity of bottle feeding (Badran et al., 2020; Jenkins et al., 2023). Over 50% of these infants attained full oral feeds within 2 weeks. Many of the infants who received a G-tube (non-responders) were infants of diabetic mothers (IDMs) who had poor glucose control during pregnancy. In fact, 8 out of 9 infants of poorly controlled diabetic mothers failed to respond to taVNS alone, while 75% of all IDMs, including those exposed to gestational diabetes, required a G-tube for hospital discharge.

IDMs are at risk of feeding delays related to intrauterine hyperglycemia and CNS oxidative stress, resulting in lower brain-derived neurotrophic factor (BDNF), changes in white matter structure, and smaller global and regional brain volumes that persist into adolescence (Rizzo et al., 1995; Teodoro et al., 2013; Bathina et al., 2017; Durga et al., 2018; Huerta-Cervantes et al., 2020; Luo et al., 2023). Exposure to prolonged hyperglycemia *in utero* results in *continuing* CNS oxidative stress postnatally and inhibition of the cortical neuronal plasticity required for learning (Escobar et al., 2013; Teodoro et al., 2013; Satrom et al., 2018; Franzago et al., 2019; Huerta-Cervantes et al., 2020). With known impairment of synaptic plasticity, exposure to intrauterine hyperglycemia carries the potential for delays in developing motor skills, such as coordination of more than 30 muscles required for effective feeding (Ornoy et al., 2001; Matsuo and Palmer, 2008; Camprubi Robles et al., 2015; Satrom et al., 2018). With magnetic resonance spectroscopy (MRS) obtained before and after the taVNS treatment period, we showed that reduced glutathione (GSH) concentrations in the basal ganglia, a measure of CNS oxidative stress, predicted response to taVNS-paired feeding only in the IDMs (Jenkins et al., 2023). Neuroplastic changes were evidenced by increased complexity and microstructure of specific white matter (WM) tracts in diffusion kurtosis imaging (DKI) and were significantly greater in infants who attained full oral feeds during taVNS-paired feeding than in those who needed a G-tube for feeds (Jenkins et al., 2023). Taken together, these data suggest that there were blocks to neuroplasticity in those who did not improve, half of whom were IDMs. Furthermore, persistent oxidative stress had an impact on learning this motor skill in the IDMs and may have been responsible for the potential block to neuroplasticity with taVNS.

N-acetylcysteine (NAC) is an antioxidant and GSH precursor that increases CNS [GSH] when given intravenously (IV) in infants (Moss et al., 2018; Jenkins et al., 2021) and is FDA-approved in children of all ages. We have previously shown dose-responsive increases in [GSH] in the basal ganglia that positively correlated with plasma [NAC] after IV dosing in infants with HIE (Moss et al., 2018; Jenkins et al., 2021). However, there are no published pharmacokinetic (PK) or bioavailability data with enteral dosing of NAC in infants and no pharmacodynamic (PD) evidence of GSH target attainment, all of which are essential to rigorously determine if enteral NAC dosing is adequate to change the oxidative milieu in the CNS. The NAC bioavailability of 6–10% reported in adult healthy volunteers is thought to be due to extensive first-pass metabolism of NAC in the gut wall and liver (Borgstrom et al., 1986), and neonates have decreased first-pass metabolism of drugs, which may lead to greater bioavailability. Therefore, in this proof-of-concept pilot trial, we hypothesized that treating oxidative stress with NAC before starting taVNS may mitigate oxidative stress and enable taVNS to boost neuroplasticity and enhance motor learning, resulting in improved oral feeding in IDM preparing for a G-tube, compared to our prior cohort of IDMs receiving taVNS alone.

## 2 Materials and methods

### 2.1 Study overview

This prospective, open-label study was conducted at the Medical University of South Carolina, approved by the Institutional Review Board, conducted according to the Declaration of Helsinki, and registered on Clinicaltrials.gov (NCT04632069). With parental consent, we enrolled 10 IDMs >39 weeks post-menstrual age (PMA) at enrollment, who were clinically stable, on minimal respiratory support (nasal cannula or room air), and were determined by the clinical team to be G-tube candidates. We included any IDM failing oral feeds if there was a diagnosis of maternal diabetes, as glucose control (HgbA1c) during pregnancy may be unknown in some infants transferred into a tertiary care center or in non-compliant diabetic mothers. Exclusion criteria were infants with major unrepaired congenital anomalies or anomalies that limit feeding volumes, cardiomyopathy, or repeated episodes of autonomic instability (apnea or bradycardia) that were not self-resolving. In our prior study (Badran et al., 2020), we used the same inclusion and exclusion criteria without a specific provision for IDM status. In that cohort, we enrolled 12 IDMs who received taVNS alone and who largely did not reach full oral feeds (9/12). Analysis of data from that IDM subgroup formed the impetus for this clinical trial.

Our primary outcomes were safety, NAC plasma pharmacokinetics, and CNS [GSH] pharmacodynamic changes with nasogastric-administered NAC. Our primary feeding outcomes were changes in daily oral (PO) feeding volumes before and during taVNS within the treatment group and during treatment between the current cohort of NAC + taVNS and our previous IDM cohort receiving taVNS alone. We also compared the categorical number of infants who achieved full oral feeds in the NAC + taVNS group compared to our prior IDM cohort, who only received taVNS.

**Figure 1:**
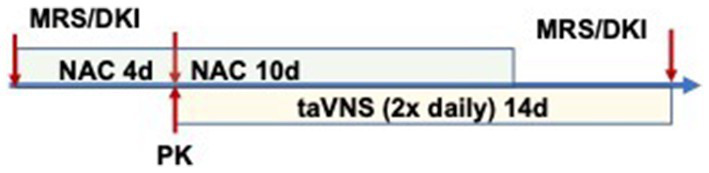
Study procedure timeline.

Study procedures were performed as outlined in Figure 1, with MRS before NAC, after 3–4 days of NAC (before taVNS initiation), and at the end of the study. NAC dose was given 2 h before the MRS scan on days 3–4 of NAC, and we obtained simultaneous plasma for determining concentrations of plasma NAC and CNS GSH. taVNS was administered for 14 days, 10 of which were with NAC administration (Figure 1).

### 2.2 Participants

We enrolled 10 infants who had diabetic mothers (Types 1, 2, or gestational DM), 9 of whom had poor glycemic control during pregnancy, defined by elevated hemoglobin A1c (HgbA1c), diabetic ketoacidosis, or obstetrical history if HgbA1c was not available. Enrolled participants were clinically determined to be failing oral feeds and were being considered for G-tube placement to achieve oral feeds sufficient for discharge from the hospital.

### 2.3 NAC administration protocol

Enteral NAC dosing was determined by applying plasma NAC pharmacokinetic and CNS GSH pharmacodynamic data from our previous trial in hypoxic–ischemic encephalopathic infants (Moss et al., 2018; Jenkins et al., 2021) and the likelihood of higher bioavailability in infants compared to adults. NAC was dosed at 75 mg/kg by nasogastric tube every 6 h (*n* = 4), or 100 mg/kg/dose every 6 h (*n* = 6), administered 1 h before a feed for a total of 14 days. All infants had existing nasogastric tubes for administering feeds as they were not taking all feeds by mouth. The 75 mg/kg dose was diluted 1:3 with sterile water. The 100 mg/kg dose was diluted 1:2 with sterile water to reduce dose volume with an increase in dilution to 1:3 if not tolerated. We monitored for emesis with the NAC administration. We followed standard clinical practice by giving a sterile water flush of 1 mL before and after the NAC dose to clear the nasogastric tube.

### 2.4 taVNS-paired feeding protocol

We delivered taVNS twice daily during bottle or breastfeeding, timing stimulation with observed sucking and swallowing, and pausing stimulation during rest or burping. The feed duration was a maximum of 30 min. The taVNS treatment period was 2 weeks, with the possibility to continue for an additional week if substantial progress was made, but full oral feeds were not yet attained. If PO feeds had not progressed after 2 weeks of taVNS treatment, the parents and the clinical team made decisions about the timing of the G-tube placement.

#### 2.4.1 taVNS parameters

**Figure 2:**
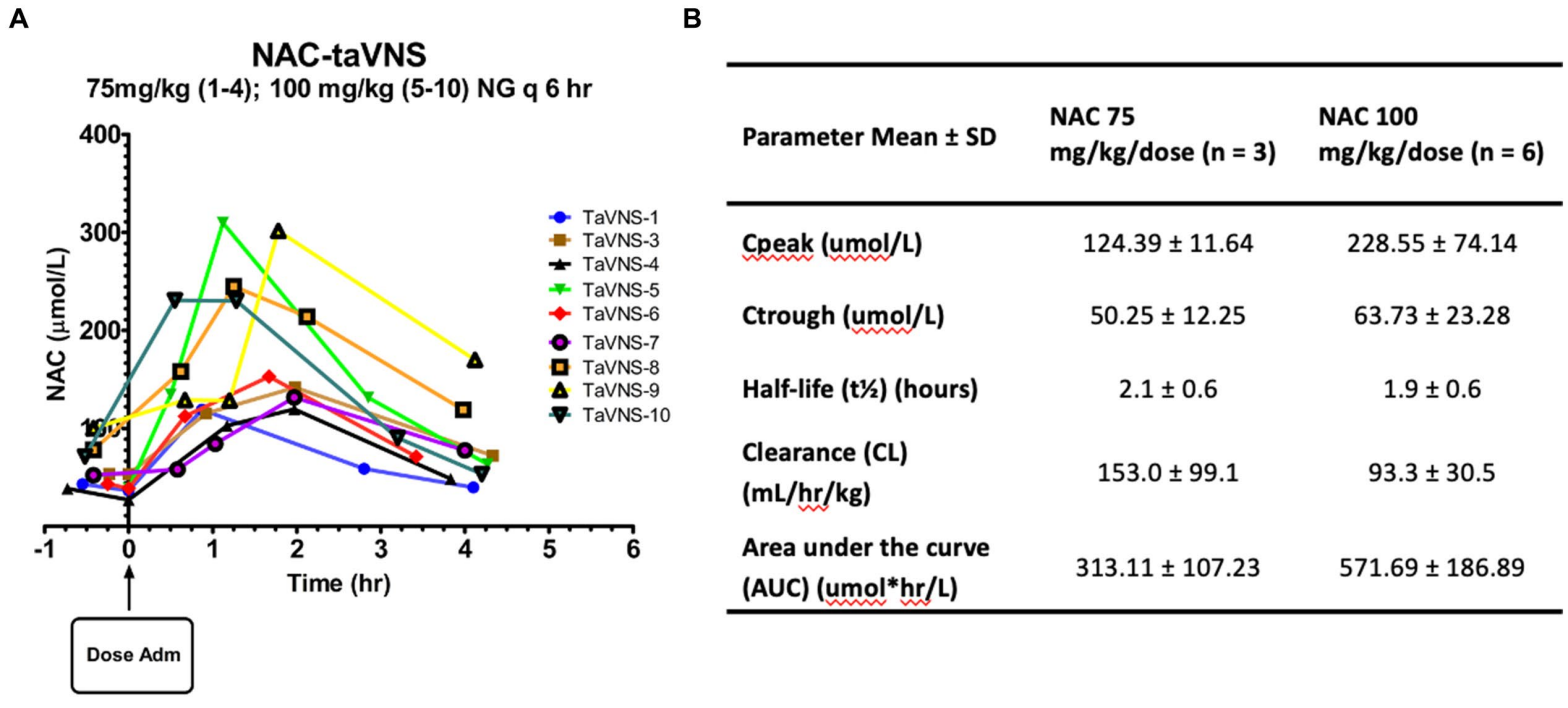
Pharmacokinetics of enteral NAC in infants: **(A)** Plasma NAC concentrations versus time after dose curves for individual participants. **(B)** PK parameters for NAC administered by NG q6h.

We delivered taVNS using a constant current electrical nerve stimulator (Soterix Medical Inc., Woodbridge, NJ) connected to a custom-designed neonatal ear electrode (anode placed at the inner tragus) and Micro Neolead electrocardiograph electrode (Neotech Products, LLC, Valencia, CA) as the cathode on the outer tragus (Figure 2). Stimulation was triggered manually and timed with individual suck bursts with a 10-s train or by continuous cycling for 2 min if sucking and swallowing became more consistent. Stimulation parameters consisted of frequency 25 Hz, pulse width 500 *μ*s, and current intensity 0.1 mA below perceptual threshold (PT). We determined PT by increasing the stimulation current in 0.1 mA increments while monitoring for indications that the infant perceived the stimulation, indicated by shrugging, change in facial expression, or fidgety movements. A neonatologist or research assistant performed the stimulation. The taVNS administration parameters were identical between this and the prior trial, but we used the Digitimer electronic pulse generator (Digitimer DS7A, Digitimer LTD) or the Soterix taVNS EPG in the prior trial (Badran et al., 2020; Jenkins et al., 2023). During treatment, the infants were fed by occupational or speech therapists, staff, or parents. We recorded PO volume intake during the taVNS feed, total daily PO volume, and any adverse events.

### 2.5 Pharmacokinetic studies

Blood samples (0.5 mL) were collected by heel stick at baseline and steady state before and at 0.5, 1, 2, and 4 h after the dose on days 3–4 of NAC, around the MRS. The plasma was frozen at −80°C until assayed for NAC concentrations (Jenkins et al., 2021). The MRS scan was timed to occur within 1.5–2.5 h after the NAC dose was administered. Exact times for blood sampling and MRS were recorded for pharmacokinetic analysis. Total plasma NAC concentrations were determined using a modified, sensitive, and specific high-performance liquid chromatography method as previously described (Wiest et al., 2014; Jenkins et al., 2016; Moss et al., 2018). Peak and trough steady-state NAC concentrations (Cpeak, Ctrough) were determined for each participant. Based on our previously reported relationship between plasma [NAC] and change in basal ganglia [GSH] in HIE infants (Moss et al., 2018), our target peak steady-state concentration (Cpeak) plasma [NAC] was 245 ± 92 umol/L.

Non-compartmental pharmacokinetic modeling (PK Solutions 2.0; Summit Research Services, Montrose, Colorado) was used to analyze plasma NAC concentration-time curves and determine the terminal elimination half-life (t1/2), volume of distribution (Vd), total body clearance (CL), and area under the concentration curve (AUC).

### 2.6 Neuroimaging

We obtained MRS and DKI data at baseline before starting NAC, after 4 ± 1 days of NAC, within 2.5 h of a regularly scheduled NAC dose, and at the end of the taVNS treatment period. Infants were fed immediately before the MRI and swaddled in a MedVac vacuum MRI immobilization system (Contour Fabrications, Inc., Fenton, Michigan) to induce natural sleep without sedation. Scans were obtained on a 3 T Magnetom Skyra or Vida MRI scanner (Siemens Healthineers, Erlangen, Germany) as previously published (Moss et al., 2018). In brief, MRS sequences STEAM TE 20 ms and PRESS TE 270 ms were performed with voxels placed in the left basal ganglia (BG) and right frontal white matter, including water reference scans for absolute metabolite concentration. Metabolite concentrations were quantified using LCModel (Moss et al., 2018, 2019), and scans were excluded if they had poor quality, as described previously (Moss et al., 2018). There were inadequate numbers of post-treatment DKI scans available in this cohort to include in this report.

For determining the CNS pharmacodynamic effects of NAC, we measured GSH by MRS (Moss et al., 2018). NAC provides cysteine, the rate-limiting precursor for GSH synthesis. CNS GSH, therefore, represents NAC’s ability to increase antioxidant concentrations in the target tissue and have a pharmacodynamic effect. With adequate NAC plasma concentrations, we have shown significant increases in CNS GSH within 12–30 min after intravenous NAC administration (Moss et al., 2018), but CNS GSH changes with enteral NAC have not been described in infants.

### 2.7 Safety monitoring and target engagement

We monitored emesis for an indication of gastrointestinal upset with NAC. Per our previous protocol, the neonatal and infant pain scale scores (Lawrence et al., 1993; Witt et al., 2016) were recorded at initiation, midway, end, and 5 min after each treatment session. If scores increased greater than 3 points or the infant appeared to be sensing the stimulation, we decreased the current intensity by 0.1 mA. We monitored redness and skin irritation at the electrode site and heart rate on bedside monitors for bradycardia, defined per nursery protocol as <80 beats per min for 5 s. For target attainment, we monitored the lowest heart rate within the first 60 s of stimulation and noted the rebound heart rate to verify the target engagement of the vagus nerve using the parasympathetic response as an indicator (Badran et al., 2018). We checked and adjusted the electrode placement if a heart rate decrease was not observed.

### 2.8 Primary outcome measures

The primary safety outcomes of this study were emesis and bradycardia during taVNS feed. The primary clinical outcome was the increase in oral feeding volumes from before NAC and during NAC + taVNS, as well as a binary endpoint of full oral feeds or the requirement for G-tube implantation. Responders were participants who were able to increase and maintain full daily PO intake (≥ 120 mL/kg/d) and gain weight adequate for discharge (≥ 20 g/day). Infants who had inadequate intake during taVNS treatment were classified as non-responders. The pharmacokinetic and pharmacodynamic outcomes were a peak plasma NAC concentration within our target range and an increase in CNS [GSH], respectively.

### 2.9 Statistical analyses

#### 2.9.1 Sample size

Our proposed sample of 10 IDMs would allow us to detect moderate effects on the feeding volume and [GSH] outcomes due to adding NAC to taVNS. For the change in daily PO feeding volumes: Our prior IDM cohort (*n* = 12) had an increase of 0.2 mL/kg/d in PO feeding volume over 10 days before taVNS versus 2.3 mL/kg/d during taVNS (similar to non-responders). Responders who attained full oral feeds had an increase of 3.8 mL/kg/d in PO volumes. Five IDM responders (50% attaining full oral feeds) would allow us to detect a significant difference between before and during NAC + taVNS (power 80%, a = 0.05, paired *t*-test).

Our target PD goal was to administer an NAC dose that would result in a consistent BG [GSH] increase, as previously published (Moss et al., 2018). In our prior taVNS trial, our mean baseline BG [GSH] in IDMs was 1.74 ± 0.23 mM, while normal CNS [GSH] is ~2 mM in newborns (Kreis et al., 2002). With 80% power and alpha of 0.05, we would need 7 patients to show a significant change in basal ganglia [GSH] of 0.14 ± 0.13 mM before and after NAC (paired t-test). Our enrollment of 10 patients allowed for the loss of MRS data due to motion or poor quality.

#### 2.9.2 Data analysis

Behavioral feeding data were analyzed via repeated measures of linear regression with the individual participant’s daily PO volumes as the dependent variable and time and treatment group (NAC + taVNS vs. taVNS alone) as independent variables. The time periods analyzed were (1) 14 days before taVNS and (2) 4 days of NAC alone followed by 1–14 of NAC + taVNS-paired feeding period (Prism 10, GraphPad Software). We included the 4 days of NAC alone with the NAC + taVNS period, as NAC is part of the treatment protocol and may have independent effects on attention and learning (Costa et al., 2016; Skvarc et al., 2017). We compared slopes of change in feeding volumes over the two time periods before and during treatment within IDM groups (NAC + taVNS or within the prior IDM cohort treated with taVNS alone) and between groups before treatment and during treatment (NAC + taVNS vs. IDM treated with taVNS alone). For differences in the numbers of IDM attaining full oral feeds between NAC + taVNS and taVNS alone IDM groups, we used Fisher’s exact test with a 2 × 2 contingency table.

For MRS metabolites, we performed paired *t*-tests to determine differences before and after treatment within the group. For taVNS parameters and heart rate changes, we used unpaired t-tests between groups.

## 3 Clinical characteristics and taVNS parameters

We enrolled 10 IDMs, all of whom were born between 30 and 37 weeks post-menstrual age. Demographic data are shown in Table 1. Poor glycemic control during pregnancy was indicated by maternal HgbA1c for 6 out of 10 mothers and by history and failed glucose tolerance tests for 2 mothers who did not have HgbA1c values and did not get glycemic treatment. One mother presented with diabetic ketoacidosis immediately before delivery, and one mother had good glycemic control at delivery with her type 1 DM. Sixty percent of IDMs had CNS insult of intraventricular or cerebellar hemorrhage, white matter infarction, or moderate/severe HIE and seizures. Two infants underwent therapeutic hypothermia for HIE for 72 h after birth. Four infants also had genetic abnormalities (duplication of 22q11.2, SMARCC2 transcription factor variant, significant homozygosity, and ARID1A gene mutation), and 4 were exposed to an intrauterine infection or maternal systemic infection with COVID-19. Perinatal infection and inflammation are independently associated with white matter neuroinflammation and infarction and worse neurodevelopmental outcomes (Alshaikh et al., 2013; Bakhuizen et al., 2014; Bright et al., 2017; Dubner et al., 2019). Eight out of 10 infants had significant gastroesophageal reflux requiring treatment with a histamine-2 receptor antagonist or proton pump inhibitor.

**Table 1:** Infant clinical characteristics for IDM poor feeders treated with NAC + taVNS or taVNS alone (1).

IDM poor feeders	NAC + taVNS (*n* = 10)	taVNS alone (ref 1) (*n* = 12)	*p*
Sex M/F	6/4	3/9	
Mean PMA at birth (weeks)	34.2 ± 2	32.3 ± 4.3	ns
Mean birth weight (grams)	2,960 ± 971	2,161 ± 1,050	ns
Mean PMA at enrollment (weeks)	41 ± 1.1	41.5 ± 2.9	ns
Mean days attempting PO before taVNS	38 ± 9	36 ± 11	ns
Mean maternal HgbA1c*	8.1 ± 2.3*	11.0 ± 2.5*	ns
Sepsis (including NEC, pneumonia, UTI, viral infections)	6	6	
CNS abnormalities (number subjects)	6	9	
IVH or other intracranial	5 (gr 1–2; & parieto-occipital lobes, cerebellum)	5 (gr 1–4, & cerebellum)	
HIE	4	2	
White matter infarction or PVL	3	2	
Lenticulostriate vasculopathy	2	1	

PMA, post-menstrual age; HgbA1c, hemoglobin A1c; HIE, hypoxic–ischemic encephalopathy; IVH, intraventricular hemorrhage; NEC, necrotizing enterocolitis; PVL, periventricular leukomalacia; UTI, urinary tract infection. *HgbA1c values available in 7 NAC + taVNS and 4 taVNS participants.

At enrollment, infants were of term post-menstrual age or older (Table 1). Oral feeds had been attempted for a mean (SD) of 38 ± 9 days before study enrollment. From previous data at our institution, >90% of preterm infants have attained full PO feeds by 40 weeks post-menstrual age and 30 days of attempting PO feeds (Gehle et al., 2022). Prior to enrollment in this research trial, the clinical team had approached parents about the need for a G-tube.

taVNS parameters of mean current intensity and duration of a treatment session and decrease in heart rate at onset of stimulation (determining the perceptual threshold) are detailed in Table 2 for both the NAC + taVNS and taVNS alone IDM cohorts (Table 2).

**Table 2:** Mean taVNS parameters and effects on heart rate in IDMs treated with NAC + taVNS or taVNS alone (mA milliamperes; *heart rate change occurred within 30 s of starting stimulation, with a return to baseline within 60 s; unpaired *t*-test significance *p* < 0.05).

IDM poor feeders	NAC + taVNS (*n* = 10)	taVNS alone (ref 1) (*n* = 12)	*p*
Perceptual Threshold in mA (mean, SD)	1.41 ± 0.43	0.9 ± 0.23	<0.0001
Stimulation Current Intensity in mA (mean, SD)	1.14 ± 0.49	0.78 ± 0.23	<0.001
Duration of individual treatments (mean, SD)	26 ± 2	28 ± 3	ns
Number of treatments (mean, SD)	26 ± 9	24 ± 12	ns
Heart rate change from baseline* (mean, SD)	−18 ± 2	−15 ± 4	0.04
Lowest heart rate during stimulation	134 ± 12	130 ± 11	ns

Most stimulation sessions were performed with bottle feeding, but in two mothers, taVNS-paired feeding treatments were also conducted during breastfeeding, and these infants were able to successfully breastfeed by discharge.

### 3.1 Safety

There were no adverse bradycardia events during taVNS-paired feeding. No increase in emesis was reported with the NAC 75 mg/kg/dose (1:3 dilution). Two patients receiving 100 mg/kg/dose (1:2 dilution) had increased emesis, which resolved when the dilution was increased to 1:3 for subsequent participants.

### 3.2 Pharmacokinetics

Individual NAC plasma concentration-time curves are shown in Figure 2A. We started at an initial dose of 75 mg/kg/dose every 6 h while monitoring closely for adverse drug reactions and analyzing NAC plasma concentrations after each patient. After the first four patients, we recognized the need for a higher NAC dose due to the resulting low plasma concentrations. The NAC dose was subsequently increased to 100 mg/kg/dose every 6 h using a 1:2, then a 1:3 dilution to minimize dose volume without emesis. Mean PK parameter estimates for the two dose groups can be found in Figure 2B. One patient could not be included due to insufficient blood sample volumes. Steady-state NAC plasma concentrations with the higher 100 mg/kg/dose every 6 regimen were within our target range, which was based on the relationship between plasma [NAC] and BG [GSH] in our prior study of HIE infants (Jenkins et al., 2021). The increase in NAC dose from 75 to 100 mg/kg/dose resulted in a proportional 26% increase in trough concentration at steady state (Ctrough). However, there was an 84% increase in peak concentration (Cpeak) at steady state and an 83% increase in the area under the curve with the higher dose, which indicates a non-linear response, partially explained by the 40% decrease in clearance but could also be related to higher bioavailability. We were not able to calculate bioavailability directly due to the lack of IV dose comparison, along with the high variability in area under the curve and clearance in the 75 mg/kg/dose group and the non-linear response in the 100 mg/kg/dose group.

### 3.3 GSH concentrations in the basal ganglia

**Figure 3:**
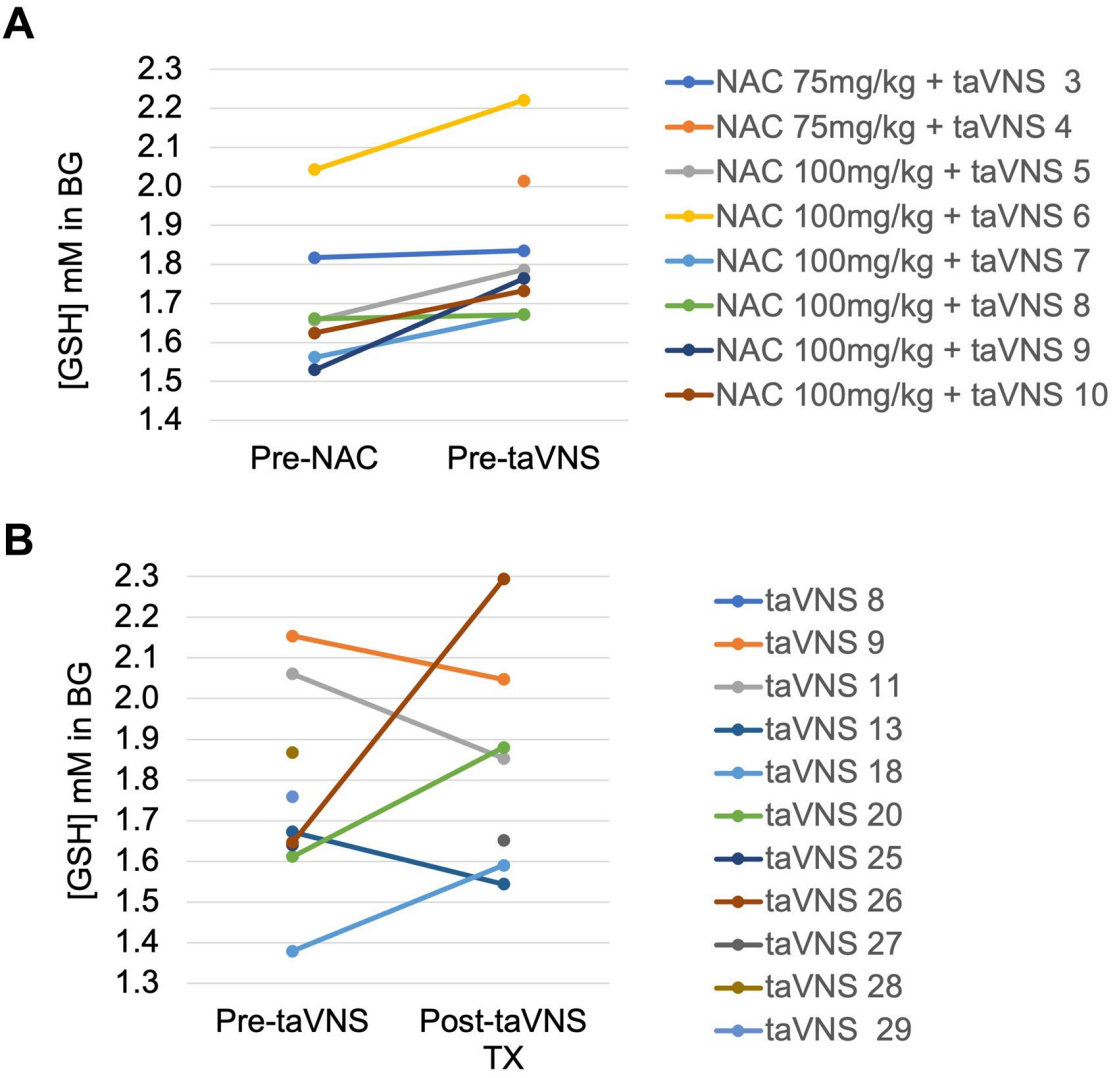
Change in reduced glutathione in the basal ganglia in IDMs: **(A)** Before and after 4 days of enteral N-acetylcysteine in the NAC + taVNS group. **(B)** Before and after 2 weeks of taVNS alone.

[GSH] in the basal ganglia consistently and significantly increased from before to after 3–5 days of NAC (mean + 0.13 ± 0.08 mM) with NAC 100 mg/kg/dose NG (*p* = 0.01, Figure 3A). MRS data were insufficient to analyze in the NAC 75 mg/kg group due to technical errors (*n* = 2) and motion-degraded spectra (pre-NAC scan for participant 4) and for the third scan in participants due to lack of availability of the in-patient clinical scanner for three infants before discharge from the hospital on full feeds. In the taVNS alone cohort, the first scan was performed 1–2 days before taVNS start (*n* = 10) and the second scan at the completion of 2 weeks of taVNS treatment (*n* = 7, Figure 3B). There was no consistent change in [GSH] with taVNS alone and wide variability, mean (SD) 0.1 ± 0.33 mM, *p* = 0.4 (Figure 3B).

### 3.4 Feeding outcomes

Seven infants (4 received 75 mg/kg NAC and 3 received 100 mg/kg NAC) attained full oral feeds with adequate weight gain during NAC + taVNS-paired feeding in a mean of 14 ± 8 days. This 70% response rate for NAC + taVNS is higher than the 25% response rate for the taVNS alone IDM cohort (*p* = 0.034, Fisher’s exact test). In one infant, nurses and therapists fed the baby full PO feeds for 5 days, but the teen mother was unable to effectively feed the infant, and the grandmother was unavailable to help for much of the day. This full PO-feeding infant, therefore, required a G-tube for discharge along with three other infants who did not reach full oral feeds. Another mother elected to have a G-tube placed post-discharge when her infant developed fungal esophagitis. He was a twin and the 10th child to this mother, who was overwhelmed and worried about her ability to handle him with this condition at home. These cases reinforce the complex interplay of home and environmental factors in successful infant feeding, even after oromotor proficiency is attained.

Time was a significant determinant of feeding volumes during treatment (*F* = 104 taVNS alone, *F* = 188 NAC = taVNS, both *p* < 0.0001), but not before treatment. We included 4 days of NAC alone as part of the treatment period in the NAC + taVNS group, as NAC may have its own effects on attention and learning (Skvarc et al., 2017).

**Figure 4:**
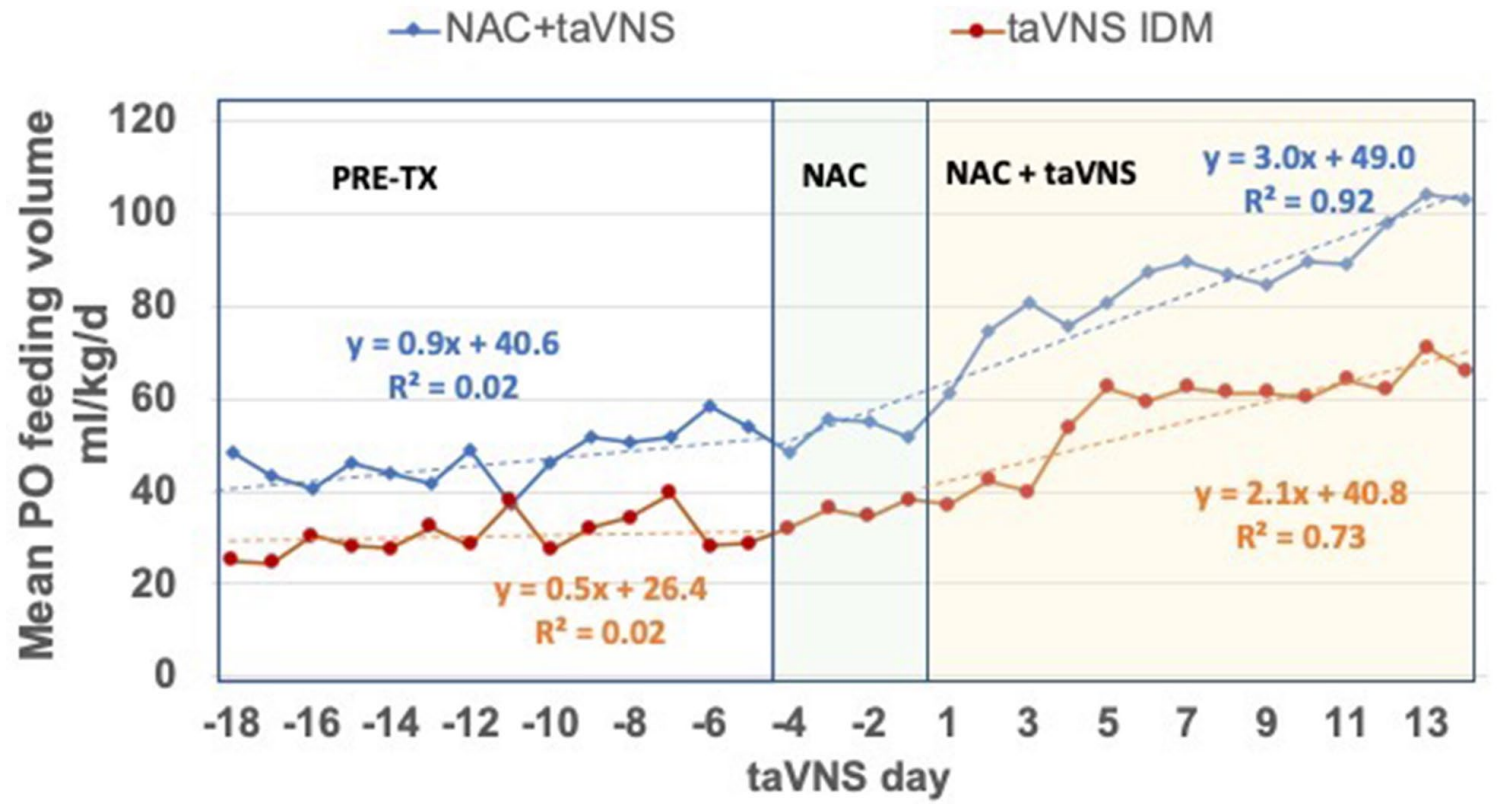
Daily PO feeding volumes in IDMs treated with NAC + taVNS vs. taVNS alone. The 14-day period before treatment (Pre-TX); and the 18-day period during 4 days of NAC alone and 14 days of NAC + taVNS, or 14 days of taVNS alone. Both groups started taVNS on day 1. Regression formulas for each group indicate the increase in PO feeding volume per day in the two periods: NAC + taVNS group (blue lines) PO volumes were + 0.9 ± 0.6 mL/kg/d pre-treatment vs. + 3.0 ± 0.4 mL/kg/d during treatment [F 7.00 (1, 316), *p* = 0.009]; taVNS alone group (red lines) PO volumes were + 0.5 ± 0.3 mL/kg/d pre-treatment vs. + 2.1 ± 0.6 mL/kg/d during treatment [F 6.19 (1, 380), *p* = 0.013]. However, the NAC + taVNS group continues to improve feeding volumes during the treatment period compared to the taVNS alone group [F 4.316 (1, 28) *p* < 0.05], which makes little progress after taVNS days 5–6.

All IDMs had a significantly greater daily increase in mean PO volumes during treatment vs. 14 days before NAC + taVNS or taVNS alone (Pre-TX, both *p* ≤ 0.01, Figure 4). Compared to 12 IDM treated with taVNS alone (Badran et al., 2020; Jenkins et al., 2023), there was a significantly greater increase in mean daily PO feeding volumes in IDM during treatment with NAC + taVNS (+3.0 mL/kg/d) compared to IDMs treated with taVNS alone (+ 2.1 mL/kg/day), [*F* = 4.32, *p* < 0.05]. The feeding volumes of IDMs treated with taVNS alone plateaued at days 5–6 of taVNS with a minimal increase during the second week of treatment, whereas the NAC + taVNS IDM group continued to increase their PO volumes throughout the taVNS treatment period (Figure 4).

## 4 Discussion

In this open-label, proof-of-concept trial, we sought to alter CNS oxidative stress, which may have been a significant contributor to the poor feeding outcome of IDMs enrolled in a prior trial of taVNS-paired feeding treatment (Badran et al., 2020; Jenkins et al., 2023). NAC rapidly crosses the blood–brain barrier and is able to reduce oxidative stress by directly scavenging oxygen free radicals and functioning as a cell-permeable source of cysteine, the rate-limiting substrate in the synthesis of the crucial intracellular antioxidant, GSH (Bavarsad Shahripour et al., 2014; Moss et al., 2018; Adams et al., 2021; Pedre et al., 2021). Therefore, in 10 IDMs failing oral feeding at term post-menstrual age and in discussions for G-tube placement, we administered enteral NAC before and during taVNS treatment. We observed that 70% of IDMs reached full oral feeds with NAC + taVNS versus 25% with taVNS alone. If confirmed in larger trials, the combination of NAC and taVNS could offer an innovative solution to feeding problems in these older IDMs, who have no other treatment options other than usual care of therapist-directed feeding.

IDMs who are exposed to uncontrolled hyperglycemia have increased oxidative stress due to the imbalance between the production of free radicals and the radical scavenging system, resulting in GSH depletion (Escobar et al., 2013; Teodoro et al., 2013; Rosa et al., 2015; Topcuoglu et al., 2015; Bathina et al., 2017). In our pilot trial of taVNS-paired feeding, CNS GSH concentrations by MRS did not independently predict non-response to taVNS but had a significant interaction with IDMs in predicting attainment of full oral feeds, strongly suggesting that ongoing CNS oxidative stress contributes to the neuropathology in IDMs failing to learn the motor skill of feeding (Jenkins et al., 2023). We thus postulated that this persistent oxidative stress in IDMs was inhibiting the neuroplasticity required to learn a motor skill and limiting any potential taVNS effect.

Hyperglycemia *in utero* also causes adverse metabolic and epigenetic changes in the perinatal period. The maternal conditions of smoking and diabetes have similar ability to cause prolonged oxidative stress that results in a persistent pro-inflammatory phenotype and increased DNA damage in the infant (Knopik et al., 2012; Franzago et al., 2019; Dincer et al., 2022). Hyperglycemia in animal models alters long-term synaptogenesis and hippocampal neurochemistry with decreased BDNF (Bathina et al., 2017; Satrom et al., 2018). In a streptozocin-induced model of gestational diabetes, oxidative stress persists for at least 2 months in rat offspring as measured by increased reactive oxygen species and decreased GSH, particularly in the cerebral cortex in males and in the hippocampus in females (Huerta-Cervantes et al., 2020). These oxidative stress markers are related closely to working memory in males and spatial learning in females (Dean et al., 2009; Bathina et al., 2017). Depletion of reduced glutathione also disrupts short-term memory necessary for encoding a complex sequence of motor skills, such as learning to suck, swallow, and breathe (Rizzo et al., 1995; Dean et al., 2009; Huerta-Cervantes et al., 2020). These pre-clinical findings of decreased BDNF and synaptogenesis, changes in neurotransmitters in the hippocampus, and correlation of reduced GSH and oxidative stress with working memory in rat pups of diabetic mothers provide potential mechanisms for both the feeding failure we observe in IDMs and the potential response to NAC + taVNS vs. taVNS alone.

IDMs who received NAC + taVNS required higher current intensity to reach their perceptual threshold (Table 2) and, therefore, received higher current intensities during stimulation than IDMs who had received taVNS alone. The reason for this observation is not clear. NAC may modulate glutaminergic synapses through metabotropic glutamate receptors and so may affect sensitivity to external stimuli, as in autism spectrum disorder (Lee et al., 2021; Zhang et al., 2023), and directly improve attention and learning (Skvarc et al., 2017). NAC’s effect on CNS glutathione may improve the redox milieu of neural cells, changing gene expression (Hung et al., 2009) to one of less oxidative stress and favoring growth factor synthesis and synaptic plasticity (Costa et al., 2016; Yi et al., 2016). Thus, NAC may enhance the effects of neuromodulation on neuroplasticity in adult diabetics after stroke or other neuroinflammatory diseases that involve oxidative stress.

Maternal diabetes is a risk factor for neonatal stroke, brain injury, and poor neurodevelopmental outcomes (Rizzo et al., 1995; Silverman et al., 1998; Ornoy et al., 2001; Camprubi Robles et al., 2015; Rosa et al., 2015; Durga et al., 2018). In addition to HIE, the IDMs in both taVNS cohorts had various CNS injuries on MRI, including hemorrhages, infarctions, and ventriculomegaly and microcephaly, indicating neuroinflammation and/or ischemia *in utero*. Even exposure to gestational diabetes carries a risk for smaller brain volumes overall and in specific gray matter regions (smaller superior temporal and bilateral rostral middle frontal gyri) in IDMs at 9–10 years in a large Adolescent Brain Cognitive Development (ABCD) Study® (Luo et al., 2023). Similar to outcomes in neonatal hyperglycemic rat models (Bathina et al., 2017; Satrom et al., 2018; Dincer et al., 2022), IDM status is associated with functional impairments of poor memory and motor and cognitive deficits in school-age children (Rizzo et al., 1995; Ornoy et al., 2001; Camprubi Robles et al., 2015).

While acute mitigation of oxidative stress shortly after stroke or perinatal asphyxia (HIE) is the target of current interventions, ischemic and neuroinflammatory brain injuries in infants do not readily lend themselves to acute treatment (Juul et al., 2020), as many are silent events occurring during a period of instability and not diagnosed by bedside tests. Our premise is novel in the extension of treatment to the chronic phase of recovery: oxidative stress is ongoing in infants with brain injury after exposure to hyperglycemia and may be treated with neuromodulation to augment rehabilitation and recovery.

This is the first time, to the best of our knowledge, that enteral NAC has been used to establish a dose that provides adequate plasma concentrations and a desired CNS GSH pharmacodynamic response in infants. The challenges we faced were the known low bioavailability in adults, establishing a dose volume and osmolality of the NAC solution that would be tolerated with minimal adverse effects (e.g., emesis), and the frequency of administration to maintain a pharmacodynamic response. The observed NAC half-life was shorter with faster clearance than observed values in our previous studies of IV NAC in infants (Wiest et al., 2014; Jenkins et al., 2021). With the 100 mg/kg/dose, we saw a greater than proportional increase in peak NAC concentration at steady state and AUC, suggesting a saturated metabolic pathway with decreased clearance and/or increased bioavailability at higher doses. Bioavailability for NAC would be determined by the formula AUC for nasogastric administration ÷ AUC for IV administration, assuming a constant drug clearance and a uniform distribution of drug once it reaches the plasma. We were not able to accurately determine the bioavailability of nasogastric NAC due to the lack of concurrent IV NAC administration and the limitations of blood sampling in infants. Using the IV NAC mg/kg/dose every 12h AUC data (265.7 ± 71.7) from our previous HIE trial in term infants (Jenkins et al., 2021), the nasogastric 75 mg/kg/dose every 6h NAC AUC data in this trial yields an estimated bioavailability of 63%. The accuracy of this estimate may be limited by differences in patient demographics between the groups (term HIE and late preterm to near-term IDMs) and the small sample size in this trial (*n* = 10). However, based on our NAC plasma concentrations (Figure 2), we think it is reasonable to say that bioavailability was much higher than the 6–10% observed in adults.

The NAC 100 mg/kg/dose resulted in adequate plasma concentrations to produce the desired pharmacodynamic response of a significant increase in CNS-reduced glutathione. In comparison, we observed no consistent change in GSH in our prior IDM cohort receiving taVNS alone. As with IV NAC administration, our data indirectly show the transport of NAC across the blood–brain barrier, with a significant change in GSH in the basal ganglia, an important area for sensorimotor integration and attention. IDMs treated with NAC and a taVNS-paired feeding intervention achieved a significantly greater increase in daily oral feeding volumes compared to pre-taVNS PO volumes and significantly greater PO volumes than the prior IDM cohort with taVNS alone. Seven out of 10 IDMs reached full oral feeds during NAC + taVNS intervention versus 3 out of 12 IDMs in the prior taVNS alone cohort while having similar clinical profiles. There was no clear NAC dose response for the attainment of oral feeding proficiency, but this may be an effect of a small sample size. Thus, although a small pilot trial, our data provide support for a larger efficacy trial using this novel approach of NAC to mitigate oxidative stress and to enhance taVNS effects on oromotor learning in IDMs failing oral feeds.

### 4.1 Limitations

The limitations of our study include a small sample size for exploration of dose and proof of concept and lack of a concurrent control group. However, our pharmacokinetic and pharmacodynamic data will guide dosing and effect sizes for further investigations in a randomized controlled trial.

### 4.2 Conclusion

This is the first trial to investigate the pharmacokinetics and pharmacodynamics of enteral NAC as an antioxidant and GSH precursor in infants. The infants of diabetic mothers who are failing to learn an oral feeding task show reliable NAC plasma concentrations and increased GSH in our CNS target, the basal ganglia, when dosed at 100 mg/kg/dose every 6 h by enteral administration. We demonstrate functional improvement with increased daily feeding volumes compared to the period before taVNS and compared to taVNS alone. This data substantiates our premise that mitigation of ongoing oxidative stress may increase response to taVNS paired with a motor task and supports further investigation of this novel neuromodulatory approach in neuroinflammatory brain injuries with oxidative stress.

## Data availability statement

The raw data supporting the conclusions of this article will be made available by the authors, without undue reservation.

## Ethics statement

The studies involving humans were approved by Institutional Review Board I, Medical University of South Carolina, Charleston, SC. The studies were conducted in accordance with the local legislation and institutional requirements. Written informed consent for participation in this study was provided by the participants’ legal guardians/next of kin.

## Author contributions

DJ: Writing – review & editing, Writing – original draft, Visualization, Validation, Supervision, Software, Resources, Project administration, Methodology, Investigation, Funding acquisition, Formal analysis, Data curation, Conceptualization. SG: Writing – original draft, Supervision, Software, Project administration, Writing – review & editing, Validation, Resources, Methodology, Investigation, Formal analysis, Data curation, Conceptualization. AB: Writing – review & editing, Project administration, Investigation, Data curation. JM: Writing – review & editing, Project administration, Investigation, Data curation. KB: Writing – review & editing, Project administration, Investigation, Data curation. LA: Writing – review & editing, Formal analysis, Data curation. SH: Writing – review & editing, Formal analysis, Data curation. HM: Writing – review & editing, Validation, Supervision, Software, Methodology, Investigation, Formal analysis, Data curation. BB: Writing – review & editing, Supervision, Resources, Methodology, Investigation, Conceptualization. MG: Writing – review & editing, Visualization, Supervision, Resources, Methodology, Investigation, Funding acquisition, Conceptualization. DW: Writing – review & editing, Validation, Supervision, Resources, Methodology, Investigation, Formal analysis, Data curation, Conceptualization.

## Glossary

AUC: Area under the curve
BDNF: Brain-derived neurotrophic factor
Cpeak: Peak plasma concentration
Ctrough: Trough plasma concentration
DKI: Diffusion kurtosis imaging
EDTA: Ethylenediaminetetraacetic acid, an anticoagulant
G-tube: Gastrostomy tube
GSH: Reduced glutathione
[GSH]: Concentration of reduced glutathione
HgbA1c: Hemoglobin A1c
HIE: Hypoxic–ischemic encephalopathy
IDM: Infant of diabetic mother
IV: Intravenous
ml/kg/d: Milligrams per kilogram body weight per day
mg/kg: Milligrams per kilogram body weight
mM: Millimoles
MRS: Magnetic resonance spectroscopy
NAA: N-acetylaspartate
[NAA]: Concentration of N-acetylaspartate
NAC: N-acetylcysteine
[NAC]: Concentration of N-acetylcysteine
NG: Nasogastric
PD: Pharmacodynamic
PK: Pharmacokinetic
PMA: Post-menstrual age
PO: Per orum – oral
PRESS: Point resolved spectroscopy
PT: Perceptual threshold
STEAM: Stimulated echo acquisition mode
t1/2: Terminal elimination half-life
taVNS: Transcutaneous auricular vagus nerve stimulation
TE: Echo time
TR: Relaxation time
TX: Treatment
umol/L: Micromoles per liter
